# 3D photothermal hydrogels derived from spinel CoMn_2_O_4_@MXene nanocomposites for an efficient solar-driven evaporation system

**DOI:** 10.1039/d6ra00897f

**Published:** 2026-04-07

**Authors:** Muneerah Alomar, Lamia Abu El Maati, Muhammad Sultan Irshad, Naila Arshad, Afraa Alotaibi, Naveed Mushtaq, Van-Duong Dao, Xianbao Wang

**Affiliations:** a Department of Physics, College of Science, Princess Nourah bint Abdulrahman University P.O. Box 84428 Riyadh 11671 Saudi Arabia; b Ministry of Education Key Laboratory of Green Preparation and Application for Functional Materials, School of New Energy and Electrical Engineering, Hubei University 430062 Wuhan China muhammadsultanirshad.hubu.edu.cn wxb@hubu.edu.cn; c School of Physics, Electronics and Intelligent Manufacturing, Huaihua University Huaihua China; d Faculty of Biotechnology, Chemistry, and Environmental Engineering, Phenikaa School of Engineering, Phenikaa University Hanoi 12116 Vietnam duong.daovan@phenikaa-uni.edu.vn

## Abstract

Freshwater scarcity and waterborne diseases are among the most pressing global challenges resulting from climate change and industrial expansion. Solar-driven interfacial evaporation systems (SDIEs) present a new approach that enables higher solar-to-heat and heat-to-vapor conversion efficiencies for higher evaporation rates of freshwater generation. However, sustainable evaporation also faces challenges associated with salt accumulation and heat losses to the environment and bulk water. Herein, a new class of photothermal nanocomposites (spinel CoMn_2_O_4_/Ti_3_C_2_ MXene nanosheets) is synthesized that exhibits enhanced photothermal conversion behavior. The 3D photothermal hydrogel is constructed by integrating the CoMn_2_O_4_@MXene nanocomposite into a polyvinyl alcohol (PVA) matrix, where a 3D porous architecture facilitates rapid water transport (hygroscopic value), localized heat confinement (39.7 °C), and salt rejection (3.5 wt%). The cross-linked hydrogel matrix prevents nanocomposite leaching during continuous evaporation (1.45 kg m^−2^ h^−1^) under one sun solar intensity. Evaporation performance under different salinities (3.5–15 wt%) confirmed the sustainability of the evaporator and reduced variability in evaporation rates, and effective desalination of seawater (salinity reduction: 99.98%) is demonstrated. This work provides a scalable, multifunctional platform for sustainable clean water generation.

## Abstract 2

This study reports CoMn_2_O_4_@MXene hydrogels, which promote an efficient solar-driven evaporation system (1.45 kg m^−2^ h^−1^) and salt rejection, ensuring long-term stability.

## Graphical abstract

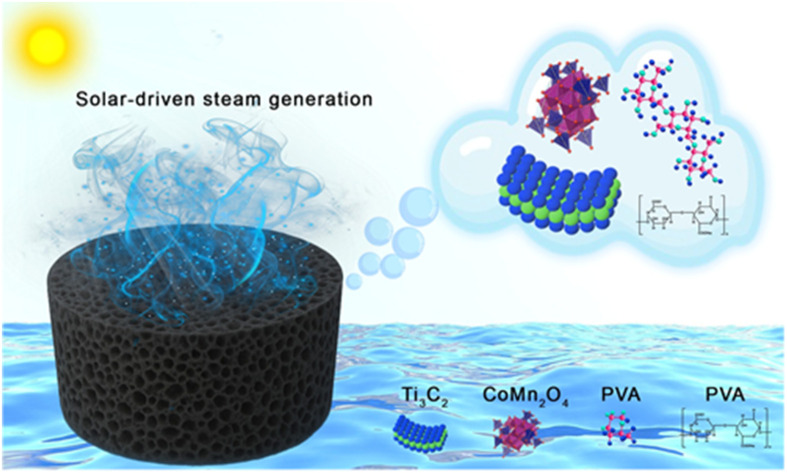

## 1 Introduction

Water scarcity is rising due to growing population and climate change. According to the United Nations, 2 to 3 billion people lack access to water for at least one month per year.^1–4^ The world produces 359 billion cubic meters of municipal wastewater annually, and about 48% of this wastewater is released without treatment, thereby polluting rivers, lakes, and coastal environments. Traditional desalination methods often require large amounts of fossil fuels to generate energy, which increases costs and environmental concerns. Solar steam generation, on the other hand, harnesses the vast amount of solar energy to facilitate the production of clean water.^5–7^ Studies show that it is especially effective in wastewater treatment and saltwater desalination, with potential energy savings of up to 60% when compared with traditional methods. Efficient harvesting of solar energy, the oldest and most accessible renewable resource, has developed through natural biological processes and is still being developed for wider applications as a clean and abundant resource.^8–11^ In order to address the underlying problems of water shortage, several effective projects have led to the development of effective solar-powered freshwater generation systems to maximize the solar-thermal conversion efficiency. As a result, solar energy has become a practical means to address the interrelated water and energy shortages while reducing environmental damage.^12–14^ However, there is still a significant gap between industrial-scale implementation and current prototypes. Limited solar absorption, inadequate thermal management, and structural failure from salt crystallization within the hydrophilic channels of solar desalination systems during operation with seawater are some of its causes.

In order to power the photothermal units, photothermal materials effectively collect incoming solar energy over the entire solar spectrum and transform it directly into thermal energy.^15–17^ Significantly, photothermal materials offer focused heat energy that enhances the charge transfer of photogenerated carriers. The sunlight absorption ability of the photothermal material plays a crucial role in determining the efficiency of the photothermal conversion process.^18–20^ In an effort to develop efficient solar-powered water desalination systems, various photothermal materials have been studied, including semiconductors, carbon-based materials, plasmonic metallic nanoparticles, and polymer-based materials.^21–24^ MXenes, especially Ti_3_C_2_, have received considerable research attention owing to their outstanding photothermal properties, such as broad-spectrum solar absorption and high thermal conductivity. According to recent research studies, MXenes in particular have been extensively studied as highly effective photothermal materials for solar steam generation (SSG) because of their superior hydrophilicity, strong broadband light absorption, and adjustable surface chemistry. To optimize interfacial water evaporation, they are widely employed in the design of hydrogels, aerogels, and porous foams.^25–28^ Despite these benefits, MXenes still suffer from a number of drawbacks, such as a significant propensity for oxidation, aggregation, and structural breakdown under oxidative and aqueous conditions, which could impact their long-term stability and performance. In this regard, Ji *et al.* established a simple silylation technique for successfully stabilising MXenes against spontaneous oxidation-induced structural breakdown and for modifying their surface characteristics with respect to hydrophilicity.^29^ Furthermore, while improvements in the evaporation rates have been the major focus of development for many previously reported SDIE systems, much less research attention has been paid to effective water collection and overall system stability, which continue to be significant obstacles for real-world desalination applications.^25,30–33^

In order to overcome these challenges, combining MXenes with functional metal oxides has been shown to be a successful method for improving material stability and photothermal performance at the same time. Specifically, spinel oxides such as CoMn_2_O_4_ have advantageous electronic structures and excellent light-absorption capabilities that enable effective solar energy harvesting through electronic transitions. These oxides can enhance the endurance of the photothermal material by improving light usage and mitigating problems such as MXene aggregation and surface oxidation when paired with MXene nanosheets. Metal oxides can provide more surface roughness and active sites, which enhance heat production and light trapping. Furthermore, the performance of solar-driven interfacial evaporation (SDIE) systems may be further enhanced by adding these nanocomposites to three-dimensional (3D) hydrogels. Hydrogel-based evaporators minimize energy loss *via* the porous 3D structure by efficiently confining heat at the air–water interface and providing a constant water supply through linked hydrophilic networks.^34–36^ Specifically, the PVA hydrogel matrix acts as a barrier that immobilizes the embedded MXene nanosheets and restricts their contact with water and oxygen, thereby enhancing the long-term durability of the photothermal material. Hydrogels are highly effective in solar-driven water evaporation owing to their unique water-rich, porous polymer structure.^23,37–40^ This structure can be considered a microscopic capillary system that can effectively transport water to the surface while confining thermal energy precisely at the evaporation interface, which can significantly reduce heat loss to the bulk water.^41–46^ Additionally, the water state in the hydrogel can also lower the energy required for vaporization, making the process more efficient than in conventional systems.

**Fig. 1:**
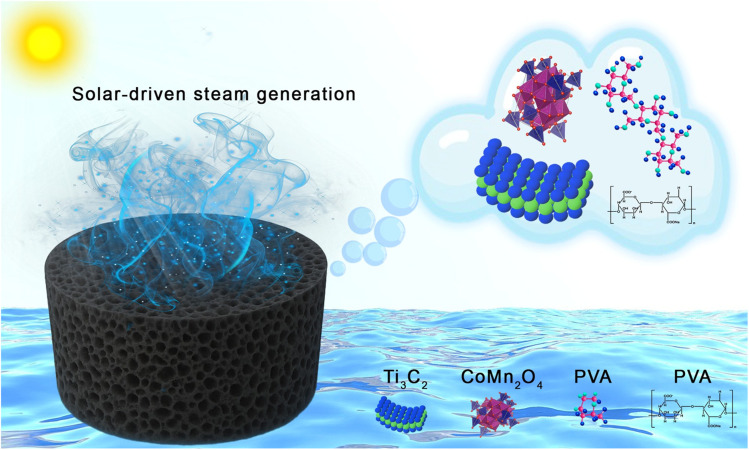
Spinel CoMn_2_O_4_@MXene-derived 3D hydrogel for efficient photothermal conversion, enabling solar evaporation for water purification and wastewater treatment.

Herein, we design a novel 3D photothermal hydrogel by embedding CoMn_2_O_4_@MXene nanocomposites into a PVA-based polymer matrix (Fig. 1). In this scheme, CoMn_2_O_4_ enhances light harvesting and strengthens the structural stability of the photothermal system, while MXene (Ti_3_C_2_) offers broadband solar absorption and effective photothermal conversion. In order to minimize thermal losses to the bulk water, these photothermal components are integrated into a PVA hydrogel matrix that creates a three-dimensional porous network. This allows for continuous water transport through capillary-driven flow, while containing heat at the air–water interface. Furthermore, during operation, the PVA framework helps prevent oxidation and aggregation and stabilizes the MXene nanosheets within the matrix. Therefore, this integrated design improves the performance of solar-driven interfacial evaporation systems by combining efficient solar absorption, better material stability, and efficient water transport pathways. This synergistic design leverages (i) the high photothermal conversion of CoMn_2_O_4_@MXene, (ii) the hydrophilic microenvironment of hydrogels for efficient water transport, and (iii) the capacity of the 3D structure for omnidirectional light capture and vapor escape. Under one sun illumination, the designed evaporator achieves a rate of 1.45 kg m^−2^ h^−1^. Additionally, it successfully removes organic pollutants and exhibits salt rejection above 99% during brine desalination. Our work advances the rational engineering of MXene-spinel nanocomposites for scalable and durable solar water purification, addressing both material stability and system-level efficiency challenges.

## 2 Experimental section

### 2.1 Synthesis of CoMn_2_O_4_ nanospheres

A precursor solution was prepared by dissolving cobalt acetate tetrahydrate (2 mmol) and manganese acetate tetrahydrate (4 mmol) in 45 mL of ethylene glycol under constant stirring. After the salts were fully dissolved, 1.5 g of hexamethylenetetramine (HMT) was introduced, and stirring was continued for an additional 30 minutes. This mixture was subsequently subjected to a 12-hour hydrothermal treatment at 180 °C within a Teflon-lined autoclave. The resulting solid was removed once the autoclave had naturally cooled to room temperature. It was then sequentially cleaned with deionized water and anhydrous ethanol before being vacuum-dried at 60 °C. A final calcination step was performed in a muffle furnace, heating the material to 600 °C at 10 °C min^−1^ and holding it at that temperature for 24 hours.

### 2.2 Spinel CoMn_2_O_4_@MXene photothermal hydrogel

CoMn_2_O_4_/Ti_3_C_2_ incorporated hydrogels were prepared *via* a freeze-drying method, combining chemical crosslinking and cyclic freeze-thaw treatments. Specifically, poly(vinyl alcohol) (PVA, MW ≈ 89 000–98 000, 99% hydrolyzed, 2.0 g) was dissolved in deionized water (10 mL) under ultrasonication (40 kHz, 30 min), followed by heating at 80 °C for 1 h to ensure complete dissolution. Glutaraldehyde (GA, 50 wt% in H_2_O, 250 µL) was then added as a chemical crosslinker to prepare a homogeneous solution (solution A). CoMn_2_O_4_@MXene powder (0.2, 0.3, or 0.4 g), sodium alginate (0.1 g), and HCl (2 M, 1.0 mL) were sequentially introduced into solution A, and the mixture was stirred vigorously (800 rpm, 10 min) before gelation at 25 °C for 2 h. The resulting gel was purified by immersion in deionized water (24 h, 25 °C), with the water refreshed every 8 h to remove unreacted residues. To enhance mechanical stability and achieve uniform porosity, the purified gel underwent three freeze-thaw cycles (freezing at −20 °C for 12 h, thawing at 40 °C for 2 h per cycle). Finally, the structured hydrogels were lyophilized (Labconco FreeZone, −50 °C, 0.05 mbar, 48 h) to obtain porous CoMn_2_O_4_@MXene hydrogels. For control experiments, pristine PVA hydrogels were synthesized identically, omitting CoMn_2_O_4_@MXene powder.

### 2.3 Controlled solar water evaporation setup

Solar-driven water purification experiments were conducted under ambient laboratory conditions (30 °C ± 2 °C, 45% ± 3% RH) using a class AAA solar simulator (PerfectLight PLS-FX300HU) calibrated to provide AM 1.5 G spectral irradiation. The CoMn_2_O_4_@MXene hydrogel (2 cm × 3 cm = 6 cm^2^) was placed afloat on simulated wastewater/seawater inside a glass beaker aligned with the irradiation focal point. The mass change of the system was continuously monitored using a high-precision analytical balance (Mettler Toledo ME204, 0.1 mg resolution) with data acquisition every 10 s. Before each experiment, a 30-minute stabilization period was allowed to confirm thermal equilibrium (<0.5% mass change over 5 min). Evaporation rates were then recorded under 1 kW m^−2^ (1 sun) illumination for 30 min. Thermal analysis was carried out using a combination of (i) infrared thermography (FTIR E4 Pro, USA) for surface temperature mapping and (ii) dual K-type thermocouples to monitor the vapor–air interface and bulk liquid with 0.1 °C accuracy. Inductively coupled plasma-optical emission spectroscopy (ICP-OES, PerkinElmer Optima 8000) was used to analyze the collected distillate and residual brine from evaporation trials in order to measure the removal of dissolved ions and pollutants. Outdoor validation experiments were further conducted under natural sunlight, replicating the same setup and measurement protocols to confirm real-world applicability.

### 2.4 Swelling ratio and hygroscopic ratio measurement

The swelling behavior of the CoMn_2_O_4_@MXene hydrogel was evaluated by recording the changes in its diameter, height, and volume before and after water uptake. The swelling ratio (*Q*_A_) was calculated by applying the following equation:1
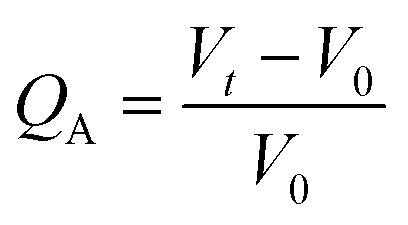
where *V*_*t*_ and *V*_0_ are the initial swollen volumes of the hydrogel, respectively. Similarly, the hygroscopic ratio (*C*_w_) of the CoMn_2_O_4_@MXene hydrogel was determined according to the following equation:2
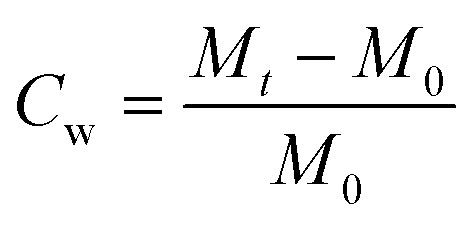
where *M*_0_ and *M*_*t*_ represent the initial dry mass and the mass of the hydrogel after water absorption, respectively.

### 2.5 Material characterization information

A field-emission scanning electron microscope (FESEM, JSM7100F, Japan) was used to determine the morphology of the sample. An energy-dispersive X-ray spectrometry (EDX) instrument was used to examine the elemental distribution within the samples. X-ray diffraction (XRD) using Cu Kα radiation was carried out using a Bruker D8 phaser device operating at 40 kV and up to 200 mA for phase structural analysis. Additionally, elemental compositions were determined by X-ray photoelectron spectroscopy (XPS) analysis utilizing a Thermo Fisher Scientific ESCALAB 250Xi system with a monochromatic Mg Kα X-ray source.

## 3 Results and discussion

**Fig. 2:**
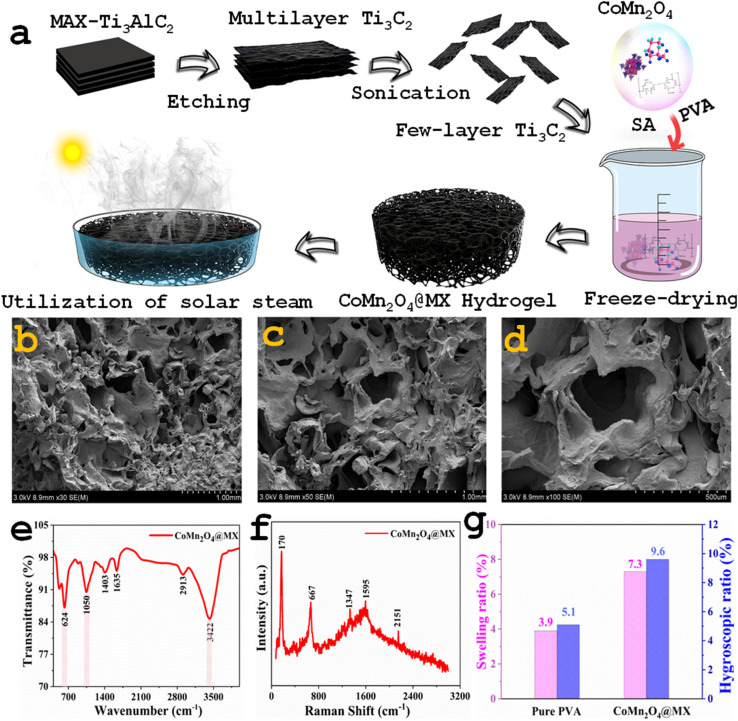
(a) Schematic of the step-by-step synthesis of CoMn_2_O_4_@MXene hydrogel. (b–d) FESEM images of CoMn_2_O_4_@MXene hydrogel showing the rough surface texture and numerous pores on the surface. (e) FTIR spectrum of CoMn_2_O_4_@MXene. (f) Raman spectrum of CoMn_2_O_4_@MXene. (g) Comparative analysis of the swelling and hygroscopic ratios of the pure PVA and CoMn_2_O_4_@MXene hydrogel.

MXene, a highly conductive 2D material, has great broadband light absorption across the spectrum of the sun and transforms it to heat with extraordinary efficiency. At the same time, the CoMn_2_O_4_ spinel oxide increases its efficiency and provides a viable option for enhanced wastewater treatment and solar desalination. The stepwise synthesis of CoMn_2_O_4_@MXene hydrogel is shown in Fig. 2a. By selectively etching away the Al layer, multilayer Ti_3_C_2_ is produced. Sonication is used to exfoliate these multilayer structures into few-layer Ti_3_C_2_ nanosheets. Next, sodium alginate (SA) and PVA are combined with CoMn_2_O_4_@MXene composite photothermal material. After freeze-drying this precursor solution, a porous CoMn_2_O_4_@MXene (Ti_3_C_2_) hydrogel is obtained. Furthermore, FESEM analysis was used to examine the microstructural morphology of the CoMn_2_O_4_@MXene hydrogel at various magnifications, as shown in Fig. 2(b–d). A three-dimensional interconnected pore structure created throughout the hydrogel is revealed in Fig. 2b, which depicts the overall porous framework of the hydrogel. Continuous water flow from the bulk water to the evaporation interface is made possible by these macroporous channels, which is crucial for maintaining effective solar evaporation. The interior pore walls and channels, which have a rough and uneven surface roughness that enhances the effective surface area and encourages improved light absorption, are more evident at a greater magnification. The pore walls and internal channels shown in Fig. 2c become more visible at a greater magnification, revealing a rough and folded surface morphology that expands the effective surface area for light absorption and evaporation. The integration of CoMn_2_O_4_@MXene nanosheets into the PVA matrix is responsible for the layered and crumpled characteristics seen in the microstructure of the pore walls, which are further highlighted in Fig. 2d. A clearly defined 3D porous network architecture with a distinctively rough surface texture is visible in the micrographs. The CoMn_2_O_4_@MXene nanocomposite exhibits effective integration and uniform dispersion within the PVA matrix which are responsible for the observed roughness. The spinel nanoparticles and the polymer chains interact with the Ti_3_C_2_ nanosheets, which are well-known for their large surface area and functional groups, resulting in a heterogeneous and complex organized surface.^47^ Because it increases the surface area accessible for light absorption and vapor escape, this textured topography is highly advantageous for photothermal evaporators. Hence, the hierarchical porous structure seen in Fig. 2b–d offers ideal circumstances for effective water transport, light harvesting, and vapor diffusion, all of which enhance the solar evaporation performance of the evaporator.

Additionally, it is evident from the images that there are many interconnecting macropores and micropores. The function of the hydrogel depends on its hierarchical porosity structure, which is a direct result of the manufacturing process.^27,48,49^ The capillary-driven movement of water from the bulk to the evaporation surface is facilitated by these linked channels, which serve as quick water transport routes.^50^ Concurrently, the porous network confines water inside the structure, reducing thermal dissipation into the underlying bulk water and efficiently localizing the converted heat at the liquid–air interface.^51^ The high rate of evaporation and the efficient thermal management properties of the material can be ascribed to the synergistic effect of the rough surface topography and the porous network. The presence of functional groups related to PVA, SA, Ti_3_C_2_, and CoMn_2_O_4_ was confirmed by the characteristic absorption bands in the Fourier-transform infrared (FTIR) spectra. Bands about ∼1625 cm^−1^ and ∼1050 cm^−1^ correspond to C=O and C–O stretching, respectively (Fig. 2e). The high hydroxyl content is responsible for the increased hydrophilicity and hydrogen-bonding interactions with water molecules, as indicated by the broad absorption spectrum between 2918 and 3422 cm^−1^. The hydrogel has higher wettability properties, allowing water to flow to the evaporative surface through the favorable adsorption sites created by the functional groups. The Raman spectrum of CoMn_2_O_4_@MXene provides additional confirmation of the structural characteristics of the composite. The D and G bands of MXene and vibrational modes of the spinel-type CoMn_2_O_4_ structure are represented by distinct peaks at ∼667, 1347, 1595, and 2151 cm^−1^. While the peaks near 1347 cm^−1^ (D band) and 1595 cm^−1^ (G band) indicate the presence of carbonaceous MXene layers with disordered and graphitic structures, respectively, the peak at approximately 667 cm^−1^ is ascribed to metal–oxygen stretching in the spinel oxide lattice (Fig. 2f). Higher-order overtones or surface interactions between the MXene sheets and CoMn_2_O_4_ nanoparticles could be the source of the extra band around 2151 cm^−1^. These findings support the hybrid integration of spinel oxide and MXene nanoparticles, which improves the structural stability and photothermal absorption of the hydrogel matrix. The CoMn_2_O_4_@MXene hydrogel exhibits better water-handling ability over pure PVA which is demonstrated by the swelling ratio and hygroscopicity analysis. The relative increase in weight following water absorption relative to its dry weight is known as the hygroscopic ratio, and it describes the capacity of a material to absorb water. It is frequently computed as follows:
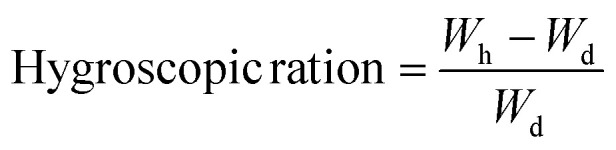
where *W*_h_ is the weight of the sample after water uptake and *W*_d_ is the dry weight of the sample. The hydrophilicity and water absorption capacity of the polymer network are reflected in the hygroscopic ratio for hydrogels. Due to the presence of hydrophilic functional groups (such as –OH and –NH), which improve the capacity of the material to absorb and hold water, a greater hygroscopic ratio is observed, which denotes a stronger connection between the hydrogel matrix and water molecules. This characteristic is especially crucial for solar-powered evaporation systems as it enhances the total evaporation performance by sustaining a steady water supply to the evaporation surface. The addition of CoMn_2_O_4_@MXene considerably raises the swelling ratio and hygroscopic ratio of the pure PVA hydrogel, which are approximately 3.9% ± 0.15% and 5.1% ± 0.25%, respectively. The high surface energy, hydrophilic functional groups, and porous structure of the composite, all of which encourage water absorption and retention, are responsible for this improvement (Fig. 2g). In addition to ensuring a quick water supply to the evaporation interface, the enhanced swelling and hygroscopic capacity also aid in preserving hydration in the face of varying solar light. In practical applications, these characteristics are essential for attaining steady and highly effective solar steam generation.

**Fig. 3:**
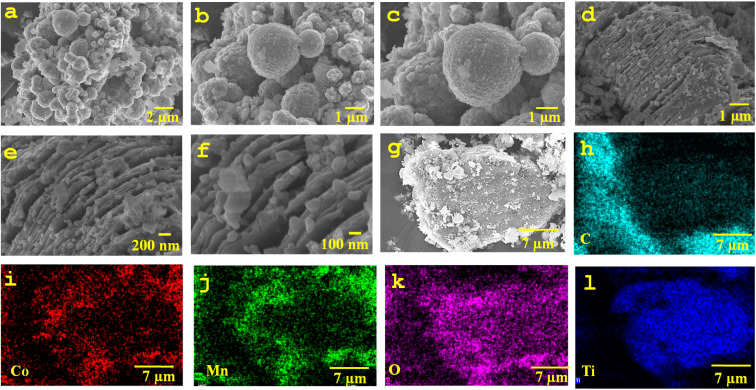
(a–c) FESEM images of spinal CoMn_2_O_4_. (d–f) FESEM images of the CoMn_2_O_4_ embedded in Ti_3_C_2_ nanosheets. (g) FESEM image and (h–l) EDS elemental mapping of CoMn_2_O_4_@MXene, affirming the presence of C, Co, Mn, O, and Ti.

MXene exhibits excellent broadband light absorption and superior photothermal conversion capacity which have made it an efficient photothermal material for solar-driven evaporation. However, there are still challenges with the oxidation and long-term stability of MXene in aqueous and oxygen-containing settings. In this study, CoMn_2_O_4_ nanoparticles were integrated into MXene, and the composite was embedded in a PVA hydrogel matrix to increase the stability of the MXene. By partially covering the MXene surface and limiting its direct exposure to water and oxygen, the CoMn_2_O_4_ nanoparticles anchored on the MXene nanosheets help prevent oxidation and structural deterioration. In the PVA matrix, the CoMn_2_O_4_@MXene nanosheets are immobilized by the cross-linked PVA hydrogel network, which also offers a protective environment that restricts oxidation and suppresses nanosheet aggregation while preserving effective water transport channels. Furthermore, the hydrophilic functional groups on the MXene surface can interact with PVA chains through hydrogen bonds, enhancing structural stability and interface compatibility. Consequently, the CoMn_2_O_4_@MXene/PVA hydrogel evaporator sustains steady photothermal performance throughout several sun evaporation cycles, suggesting that the composite hydrogel structure successfully enhances MXene stability during operation. The virgin spinel CoMn_2_O_4_ exhibits an agglomerated and spherical morphology in the FESEM images, with nanoparticles forming clusters due to high surface energy (Fig. 3(a–c)). They range in size from sub-micron to micron and feature dense, smooth surfaces. Hydrothermally generated spinel-type oxides frequently have such spherical shapes, which provide a lot of active sites for photothermal interactions. The obvious layered and sheet-like morphology can be seen in the FESEM images of CoMn_2_O_4_ embedded in MXene nanosheets (Fig. 3(d–f)). The CoMn_2_O_4_ nanoparticles are equally anchored on the surfaces and interlayers of the 2D MXene sheets, which create an interconnected layered framework. The porous and wrinkled structure of the TiO_2_ nanosheets, which offers a large surface area and plenty of anchoring sites for oxide nanoparticles, is clearly seen in the images at higher magnification (Fig. 3(e and f)).

The homogenous distribution of constituent elements in the CoMn_2_O_4_@MXene composite is confirmed by the FESEM-EDS elemental mapping (Fig. 3(g–l)), which confirms that the elements Co, Mn, O, C, and Ti are present. Since MXene delivers strong electrical conductivity, thermal transport, and hydrophilicity, and CoMn_2_O_4_ provides active photothermal sites, this homogeneous dispersion is essential for achieving synergistic effects. The CoMn_2_O_4_@MXene nanocomposite has a well-integrated hybrid structure with spherical oxide nanoparticles uniformly distributed over the MXene nanosheets and a homogeneous elemental composition, according to the combined FESEM and EDS results. This is advantageous for photothermal water evaporation because it offers superior light absorption, rapid heat transmission, and a steady supply of water within the hydrogel matrix.

The CoMn_2_O_4_@MXene hydrogel has a hierarchical porous structure which encourages numerous reflections and diffuse scattering of incident solar light within its interstitial cavities. The overall solar energy-harvesting efficiency of the material is improved by this method, which greatly increases light absorption across a wide range of wavelengths. For solar-powered steam generation systems to operate as efficiently as possible, effective thermal control is essential. The thermal conductivity of the synthesized CoMn_2_O_4_@MXene hydrogel was characterized experimentally using a Hot Disk TPS 2500 thermal constants analyzer. Upon solar radiation, a stable temperature gradient (d*T*/d*x*) is established along the vertical axis of the material.

The resultant heat flux (*q*) through the hydrogel can be quantitatively described by applying Fourier's law of heat conduction, given in the following equation:^52^3
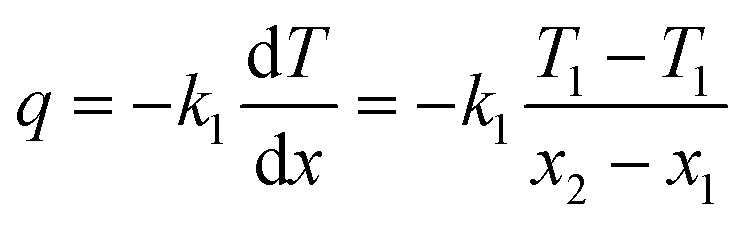


The thermal conductivity of the CoMn_2_O_4_@MXene hydrogel composite was calculated at thermal equilibrium, with the temperature maintenance rate constant. The calculation employed a steady-state heat flow equation, incorporating the established thermal conductivity of the glass slide (*k*_1_ = 1.05 W m^−1^ K^−1^), its thickness (*x*_1_ = 3 mm), and the thickness of the composite sample (*x*_2_ = 30 mm). Temperature measurements included the top surface of the conductivity meter (*T*_1_), as well as the bottom (*T*_2_) and top (*T*_3_) surfaces of the glass slides that encased the hydrogel.4
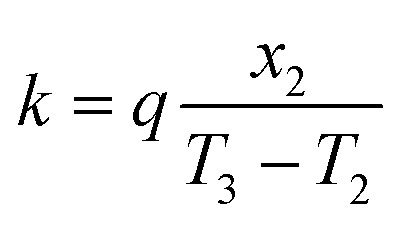


**Fig. 4:**
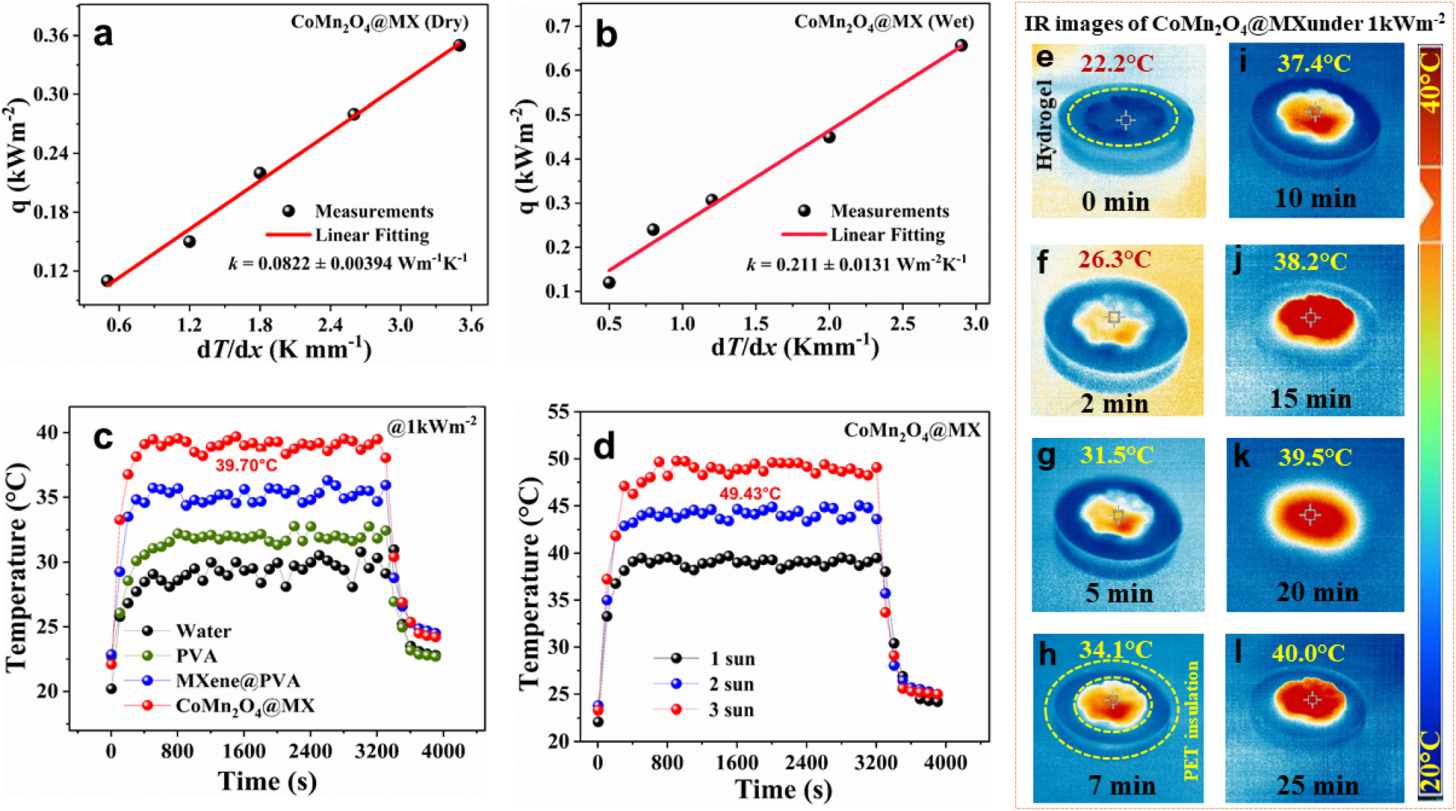
(a) Thermal conductivity measurements of CoMn_2_O_4_@MXene hydrogel (dry state). (b) Thermal conductivity measurements of CoMn_2_O_4_@MXene hydrogel (wet state). (c) Comparative surface temperature kinetics of water, PVA hydrogel, MXene@PVA composite, and the CoMn_2_O_4_@MXene nanocomposite hydrogel under 1 kW m^−2^ solar-simulated light. (d) Influence of solar flux intensity (1, 2, and 3 kW m^−2^) on the equilibrium surface temperature of the CoMn_2_O_4_@MXene hydrogel. (e–l) IR images of the CoMn_2_O_4_@MXene solar evaporator at different intervals under 1 kW m^−2^ intensity.

In the dry condition, the minimal thermal conductivity value of the synthesized CoMn_2_O_4_@MXene composite hydrogel was 0.0822 ± 0.00394 W m^−1^ K^−1^. Effective phonon scattering events at the large hierarchical interfacial surfaces of the material are responsible for this notable thermal insulation. This interior architecture facilitates the conversion of incident radiative energy into thermal energy at the photothermal interface by scattering it several times. This mechanism efficiently attenuates phonon transmission, resulting in a large drop in heat conductivity across the bulk matrix (Fig. 4a). Additionally, the hydrated thermal conductivity of the hydrogel was measured at 0.211 ± 0.0131 W m^−1^ K^−1^. This value is much lower than thermal conductivity of bulk water (∼0.6 W m^−1^ K^−1^), suggesting that even when saturated with water, the special porous structure of the composite efficiently prevents heat transmission (Fig. 4b). Optimizing interfacial surface temperature while reducing parasitic heat conduction is crucial to maximizing the effectiveness of thermal management systems. This was determined by measuring the surface temperatures of four systems, water, PVA, MXene@PVA, and CoMn_2_O_4_@MXene hydrogel, under simulated solar irradiation of 1 kW m^−2^ for one hour. Two thermocouples attached to particular areas were used to collect temperature data, as shown in Fig. 4c. The CoMn_2_O_4_@MXene hydrogel outperformed the other materials tested, showing remarkable solar energy absorption and effective lateral heat distribution inside its top matrix. By concentrating thermal energy at the air-material contact and greatly reducing downward heat conduction, this architecture facilitates superior thermal management. As a result, the photothermal surface temperature of the CoMn_2_O_4_@MXene hydrogel rose quickly to about 39.70 °C before stabilizing at an equilibrium condition. A high photothermal conversion efficiency is shown by this quick thermal reaction. At this heated contact, the underlying water, which is pulled upward through the porous network of the material, changes from a liquid to a gas, enabling effective vapor formation. Further studies focused on the connection between surface temperature and incident irradiance. The surface temperature of the CoMn_2_O_4_@MXene hydrogel rose with increasing solar flux, as seen in Fig. 4d, and reached a maximum of 49.43 °C at an irradiation intensity of 3 kW m^−2^. Accelerated steam generation rates, a key factor in determining overall system efficiency, are directly made possible by this significant temperature increase.

Infrared thermal imaging was used to track the temperature change of a CoMn_2_O_4_@MXene solar evaporator under one sun irradiation. Fig. 4(e–l) illustrates the ability of the structure to localize heat by displaying a steady rise in surface temperature over time. The initial surface temperature was found to be 22.2 °C. After being exposed to sunlight, this temperature rose to 26.3 °C in just two minutes, demonstrating the rapid thermal reactivity of the material. The temperature increases to 31.5 °C after five minutes, indicating effective heat retention and minimal energy loss.

**Fig. 5:**
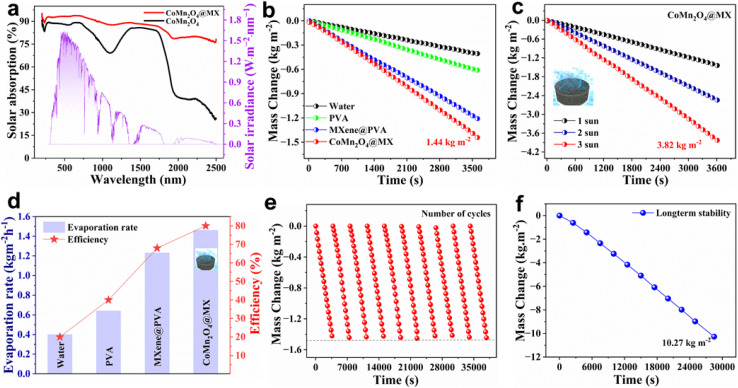
(a) UV-vis absorption spectra of CoMn_2_O_4_ and CoMn_2_O_4_@MX. (b) Time-dependent mass change per unit area for the four developed systems. (c) Mass change per unit area of the CoMn_2_O_4_@MX hydrogel under varying solar irradiation intensities. (d) Assessment of evaporation performance and solar-thermal efficiency across four different steam generation systems. (e) Evaluation of system repeatability over multiple cycles, representing the variation in measured mass change. (f) Long-term evaporation using the CoMn_2_O_4_@MX evaporator continuously over 8 h.

Efficient sun absorption is a significant aspect for photothermal materials used in solar-driven interfacial evaporation because it impacts the capacity of the material to capture solar energy and convert it into localized heat. Materials with high solar absorption may efficiently absorb energy across the whole solar spectrum, improving photothermal conversion and evaporation efficiency. To assess the capacity of the CoMn_2_O_4_@MXene photothermal material to capture light, its solar absorption spectrum was examined using UV-vis-NIR spectroscopy, employing an integrating sphere to include the entire solar spectrum (200–2500 nm). Fig. 5a shows that the CoMn_2_O_4_@MXene composite displays a higher solar absorption of ∼90% in the wavelength range of 250–2500 nm compared with pure CoMn_2_O_4_@MXene. This high absorption behavior leads to more effective light absorption and photothermal performance in solar-powered steam generation. Because of its high photothermal conversion efficiency and excellent broadband light absorption, the CoMn_2_O_4_@MXene hydrogel has a high surface temperature. This allows for localized heat concentration and ensures that absorbed solar energy is retained within the rough and porous surface texture for improved thermal efficiency. After that, the system maintains a steady, high temperature of 40.0 °C for 25 minutes. This constant performance shows that the system has reached a state of thermal equilibrium. The concentrated buildup of heat inside the upper structure enhances water evaporation by maximizing solar energy absorption. The sustained rise and sustenance of elevated temperatures support the usefulness of the CoMn_2_O_4_@MXene in augmenting solar steam generation efficiency *via* improved light absorption and heat retention.

Because of its intrinsic interfacial dispersion, which forms the basis of interfacial solar steam generation, the flux distribution across the upper surface of the CoMn_2_O_4_@MXene hydrogel facilitates remarkable photothermal conversion of incident solar radiation. The mass change of the four developed evaporators (water, PVA, MXene@PVA, and CoMn_2_O_4_@MXene) was measured over one hour under 1 kW m^−2^ solar irradiation to evaluate their comparative performance. The mass loss was quantified in units of kg m^−2^. To undertake a comparative analysis of the performance, the four systems, *i.e.*, pure water, pure PVA hydrogel, MXene@PVA hydrogel, and CoMn_2_O_4_@MXene hydrogel, were subjected to continuous sun irradiation of 1 kW m^−2^ for one hour to evaluate their cumulative mass loss per unit area and thermal efficiency. The raised surface temperature, generated by the localized surface plasmon resonance (LSPR) effect and decreased thermal conduction losses, promotes greater vapor formation. Of all the systems under study, the CoMn_2_O_4_@MXene hydrogel exhibited the greatest mass loss, at 1.45 kg m^−2^. As shown in Fig. 5b, this performance beat that of pure water (0.40 kg m^−2^), pure PVA hydrogel (0.61 kg m^−2^), and MXene@PVA hydrogel (1.20 kg m^−2^). This effectiveness is ascribed to the network of interconnected micropores that facilitates quick and continuous water transport to the evaporation surface, guaranteeing effective thermal localization and timely vapor dissipation. The interior matrix of the hydrogel simultaneously allows enough light to get through for the best possible energy absorption and subsequent conversion into thermal energy. In addition, the performance of the CoMn_2_O_4_@MXene hydrogel under increased flux was assessed at various sun irradiances. The material showed a significant mass loss of 3.82 kg m^−2^ under an irradiance of 3 kW m^−2^, as shown in Fig. 5c, confirming its improved photothermal sensitivity to higher incident energy. The photothermal conversion efficiency (*η*) of the CoMn_2_O_4_@MXene hydrogel solar steam generating system was used to measure its performance. This metric was determined using the following known energy balance principles.

Efficiency *η* is given by the ratio of the power used for vapor production to the total solar power incident on the system, as follows:5
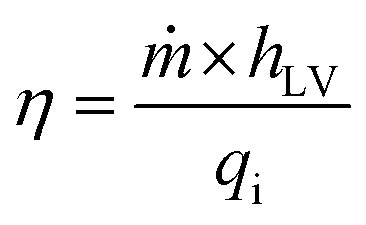
where, *ṁ* is the net mass rate of evaporation, obtained by subtracting the rate of evaporation in the dark (evaporation in the absence of light) from the rate of evaporation under solar illumination; *h*_LV_ is the total enthalpy of the liquid–vapor phase change, including both the latent heat of vaporization and the sensible heat required to increase the water temperature; and *q*_i_ is the solar irradiance (standardized at 1 kW m^−2^ for this experiment). The total enthalpy *h*_LV_ is itself calculated to include the effects of temperature variation as follows:

6 *h*_LV_ = *λ* + *C*Δ*T*

where *C* is the specific heat capacity of water (4.2 kJ kg^−1^ K^−1^), Δ*T* is the temperature increase of water, and *λ* is the latent heat of vaporization, which drops from roughly 2430 kJ kg^−1^ at 30 °C to 2256 kJ kg^−1^ at 100 °C. The investigations were conducted in a controlled setting with a temperature of 25 °C and a relative humidity of 45%. Parasitic heat losses must be taken into account in a thorough evaluation of the efficiency of the system. By taking into consideration all of the main heat loss pathways, the efficiency of the CoMn_2_O_4_@MXene hydrogel device in converting sunlight into heat was determined. These losses include the energy transferred to the surrounding water through conduction, dissipated to the environment *via* convection, and emitted as infrared radiation (eqn (7)–(9)). Thermal transport principles under one sun irradiation (1 kW m^−2^) were used to predict the performance of the system, which is governed by these photothermal processes.

Conductive heat loss is governed by the following equation:7
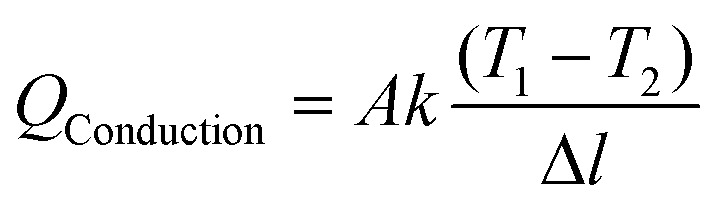
where *A* is the cross-sectional area of the evaporator, *k* is the thermal conductivity of water (0.6 W m^−1^ K^−1^), and Δ*l* is the distance between two thermocouples measuring the temperature gradient.

Convective heat loss is governed by the following equation:

8 *Q*_Convection_ = *h*(*T*_s_ − *T*_*υ*_)

In this expression, *h* is the convective heat transfer coefficient (∼10 W m^−2^ K^−1^), *T*_s_ is the surface temperature of the evaporator, and *T*_*υ*_ is the temperature of the surrounding environment, influenced by the generated vapor.

Radiative heat loss is governed by the following equation:

9 *Q*_Radiation_ = *εσ*(*T*_s_^4^ − *T*_∞_^4^)

where *ε* is the surface emissivity (estimated to be 0.93), *σ* is the Stefan–Boltzmann constant (5.669 × 10^−8^ W m^−2^ K^−4^), and *T*_∞_ is the ambient temperature. Since the vapor layer can partially transmit radiation, the actual radiative loss is bounded by calculations using *T*_∞_ equal to the ambient temperature (*T*_a_) and vapor temperature (*T*_V_), representing the maximum and minimum possible losses, respectively. Convective, radiative, and conductive heat dissipation are responsible for the overall thermal loss of about 10% of the system. From the above equations, the calculated radiative loss is around 2.8%, convective loss is approximately 5%, and conductive loss is approximately 2.2% of the incident solar energy, by taking a surface temperature of 40 °C and an ambient temperature of 25 °C. The CoMn_2_O_4_@MXene hydrogel proved to be the most effective material of the four systems studied for generating solar steam. Its performance was quantified by an evaporation rate of 1.45 kg m^−2^ h^−1^ and an efficiency of 80%, a value calculated by excluding thermal loss factors (Fig. 5d). This high efficiency positions it as a highly promising device for vapor generation. The 80% efficiency was calculated using the governing eqn (5) and (6), which incorporated the measured mass change and sample dimensions under one-sun irradiation. In order to examine the long-term stability, durability, and performance consistency of the CoMn_2_O_4_@MXene solar evaporator, consistent evaporation cycles were performed, as shown in Fig. 5e. It is expected that a solar steam generator will function well over many cycles without experiencing a significant drop in evaporation rate or structural integrity. Repeated evaporation cycles make it possible to test the long-term performance of a material under persistent sun radiation and extended water contact. The feature of a material of conducting these cycles is to discover if the CoMn_2_O_4_@MXene structure remains intact and continues to permit efficient solar absorption and thermal conversion. A continuous evaporation rate across several cycles shows that the structural stability is maintained without any degradation, blockage, or fouling, which are typical difficulties in extended water evaporation systems. The steady evaporation rate obtained over 15 cycles in the study indicates that the CoMn_2_O_4_@MXene structure retains its outstanding performance, offering a dependable and durable solution for prolonged solar-driven water purification and desalination. Fig. 5f illustrates the evaluation of the long-term evaporation stability of the CoMn_2_O_4_@MXene evaporator under constant sun illumination. Over the course of eight hours, the mass of water reduces linearly with time, showing a steady and continuous evaporation process without appreciable performance decline. This behavior demonstrates the good operational stability and durability of the evaporator during prolonged solar-driven evaporation.

**Fig. 6:**
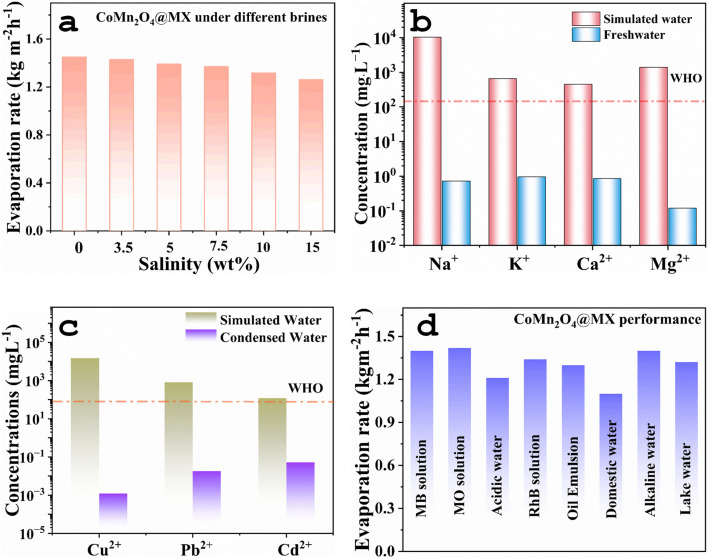
(a) Evaporation rates from the CoMn_2_O_4_@MX hydrogel under different salt concentration solutions. (b and c) Concentrations of primary and heavy metal ions, respectively, before and after operation in simulated water. (d) Evaporation rates from the CoMn_2_O_4_@MXene hydrogel under different environmental conditions.

In order to increase the evaporation rates of solar steam generators, a crucial performance indicator, significant research is ongoing to improve light absorption, thermal management, and water supply. However, a major problem for these evaporative structures is their mechanical fragility and propensity to deform in challenging operating conditions, such as excessive salinity, which significantly reduces the effectiveness of the device. In order to determine its maximum salt tolerance, the evaporation performance of the CoMn_2_O_4_@MXene hydrogel was carefully assessed under realistic settings using brine salinities ranging from 3.5 to 15 weight percent (Fig. 6a). At lower salinities, the hydrogel showed a steady rate of evaporation; however, at higher concentrations, there was a noticeable decrease because of closed water channels. This stability indicates the excellent salt-tolerance and anti-scaling ability of the hydrogel, which are critical for long-term desalination in seawater and hypersaline conditions.

A performance comparison of recently published solar-driven evaporators is presented in Table 1 to better illustrate the importance of the current study. This comparison shows how the CoMn_2_O_4_@MXene/PVA hydrogel evaporator created in this work differs from previously described systems in terms of evaporation rate, photothermal efficiency, and structural properties.

**Table 1:** Performance comparison of CoMn_2_O_4_@MXene with other recently reported evaporator designs

Evaporator design	Evaporation rate (kg m^−2^ h^−1^)	Efficiency (%)	Salt rejection performance	Long-term stability	Ref.
Porous dome array (carbonated sucrose)	3.54	95.86	Stable in 15 wt% brine (3.48 kg m^−2^ h^−1^)	Long-term stability	53
Hierarchical salt-rejection (3D-printed)	2.84	—	Stable for 7 days in 20 wt% brine and 170 h in natural seawater	7 days cyclic test	54
B4C-polyurethane architectures	1.55	—	Stable over 15 cycles under seawater	Salt-tolerance over 15 cycles under seawater	55
Fabric roll-based bionic evaporator (FRBE)	2.89	130.04	Excellent in 10 wt% and 20 wt% NaCl	Excellent salt resistance	56
MnO_2_ NWs/Chitosan hydrogels	1.72	90.6	Stable performance in multiple water media	Stable over 10 evaporation cycles	39
Large-scale 3D-printed evaporator	2.23	∼100	Fouling-resistant and decoupled design	Extends beyond traditional limits and low-cost renewal	57
CoMn_2_O_4_@MXene hydrogel	∼1.44	∼80	Stable in 15 wt% brine and in various acidic and alkaline media	Stable over 10 cycles	This work

Using inductively coupled plasma-optical emission spectroscopy (ICP-OES), the condensed water generated by evaporation was also examined to assess the nano-filtration capacity of the CoMn_2_O_4_@MXene hydrogel against common and heavy metal ions. The evaporator was quite successful in removing both primary and heavy metal ions, as seen in Fig. 6(b and c). According to the analysis, the ion content of the condensed water decreased significantly, falling well below the World Health Organization's (WHO) safe drinking water criteria. The concentrations of both primary salt ions (Na^+^, K^+^, Mg^2+^, and Ca^2+^) and heavy metal ions (Cu^2+^, Pb^2+^, and Cd^2+^) significantly decreased, according to recorded data. The solar-driven evaporation process effectively filters contaminated water, as seen by the freshwater that is produced, meeting WHO safety criteria. These results demonstrate the potential of the approach for sustainable purification, providing a way to use solar energy to create safe, clean drinking water. The CoMn_2_O_4_@MXene hydrogel was further evaluated with a variety of water sources, such as natural lake water, oil-in-water emulsions, acidic/alkaline water, dye solutions (MB, MO, and RhB), and household wastewater. All of these diverse media display good evaporation rates (Fig. 6d), highlighting the wide variety of uses and great purifying capability of the hydrogel. The consistent performance of the composite hydrogel in chemically complex and contaminated water sources indicates that it can be utilized for a variety of solar-driven water purification applications that go far beyond conventional desalination. The CoMn_2_O_4_@MXene hydrogel evaporator is therefore a great option for practical solar-powered clean water production under real-world conditions due to its exceptional salt resistance, strong ion rejection, effective heavy-metal removal, and adaptability in treating a variety of water sources.

## 4 Conclusion

In conclusion, we have successfully designed and demonstrated a robust and highly efficient 3D photothermal hydrogel evaporator for sustainable solar-driven water purification that utilizes a novel CoMn_2_O_4_@MXene nanocomposite. The nanocomposite, with a synergistic effect of enhanced broadband light absorption and enhanced photothermal conversion efficiency, was achieved by integrating spinel CoMn_2_O_4_ with highly conductive Ti_3_C_2_ nanosheets. The nanocomposite was carefully designed and synthesized within a cross-linked PVA matrix to form a 3D porous hydrogel structure, which is critical for superior performance. This structure effectively traps heat at the water–air interface (reaching a temperature of 39.7 °C under one sun illumination), promotes fast water transport through capillary action, and naturally suppresses salt concentration with proven resistance to salinity up to 15 wt%. All these factors simultaneously overcome the key hurdles in interfacial evaporation. By suppressing nanomaterial leaching and Ti_3_C_2_ oxidative degradation, the cross-linked network played a pivotal role in sustaining long-term structural integrity and enabling superior operational durability. As such, under one sun illumination, the evaporator exhibited a high and steady evaporation rate of 1.45 kg m^−2^ h^−1^. More significantly, it performed exceptionally well in desalination, lowering the salinity of synthetic seawater by 99.98% to meet drinkable water standards. As a result, this work offers a scalable, multipurpose platform that combines cutting-edge nanomaterial design with useful engineering. It offers a viable and sustainable way to alleviate the global freshwater shortage, especially in difficult settings with high-salinity water sources. Future research will concentrate on investigating large-scale, outdoor prototype applications and refining the hydrogel matrix for even greater flux.

## Conflicts of interest

The authors declare no competing interests.

## Data Availability

Data are available upon request from the authors.
